# Supplementary material for: Stinging wasps (Hymenoptera: Aculeata), which species have the longest sting?
Source: PeerJ. 2018 May 2;6:e4743. doi: 10.7717/peerj.4743 (PMC5936069; doi:10.7717/peerj.4743)
Supplement: Supplemental Information 4 — All of the code needed to run the data analyses comparing sting length to various measures. [file peerj-06-4743-s004.docx]

**Ancova comparing sting length vs mesosomal length in R.**

**Data: see file “sting_vs_size.txt”**

**R-Code:**

#overall regression does sing length predict pain

data <- read.delim("sting_vs_size.txt", header=TRUE)

attach(data)

names(data)

#regression with sting length predicted by mesosoma length and wasp type (species) and the interaction between the 2

reg2 <- lm(sting ~ mesosoma + species + mesosoma*species)

summary(reg2)

#scatter plot with points as different colors

plot(data$mesosoma, data$sting, xlab="Mesosomal Length", ylab="Sting Length", main="Sting vs Mesosomal length", xlim=c(0,15), ylim=c(0,15), cex.lab=1.4, cex.axis=.9, pch=19, col=ifelse(data$species=="w", "gray60","black"))

**Analyses of sting length vs pain**

**Data: see file “sting_data.txt”**

**R-Code:**

#read in data

data <- read.delim("sting_data.txt", header=TRUE)

attach(data)

names(data)

#raw sting lengths (not relative to body size) vs pain

reg <- lm(rawsting ~ rawPain)

summary(reg)

**Analyses of relative sting length vs pain**

**Data: see file “sting_data.txt”**

**R-Code:**

#read in data

data <- read.delim("sting_data.txt", header=TRUE)

attach(data)

names(data)

#relative sting lengths vs pain

reg <- lm(sting_ratio ~ Pain)

summary(reg)

**Analyses of relative sting length vs toxicity**

**Data: see file “toxic_data.txt”**

**R-Code:**

data <- read.delim("toxic_data.txt", header=TRUE)

attach(data)

names(data)

#relative sting lengths vs toxicity

reg <- lm(ratio ~ toxic)

summary(reg)

**Analyses of pain vs. toxicity**

**Data: see file “toxic_data.txt”**

data <- read.delim("toxic_data.txt", header=TRUE)

attach(data)

names(data)

reg <- lm(Pain ~ toxic)

summary(reg)

**Analyses of sting length vs sociality**

**Data: see file “sting_data.txt”**

**R-Code:**

#read in data

data <- read.delim("sting_data.txt", header=TRUE)

attach(data)

names(data)

reg <- lm(ratio ~ sociality)

summary(reg)

**Analyses of pain vs sociality**

**Data: see file “sting_data.txt”**

**R-Code:**

#read in data

data <- read.delim("sting_data.txt", header=TRUE)

attach(data)

names(data)

#comparing relative pain to sociality

reg <- lm(socPain ~ sociality)

summary(reg)

**Analyses of relative sting length vs host use**

**Data: see file “sting_data.txt”**

**R-Code:**

#read in data

data <- read.delim("sting_data.txt", header=TRUE)

attach(data)

names(data)

# relationship between relative sting length vs host use

reg <- lm(ratio ~ prey_graph)

summary(reg)
